# Genomic Predictions Using Low-Density SNP Markers, Pedigree and GWAS Information: A Case Study with the Non-Model Species *Eucalyptus cladocalyx*

**DOI:** 10.3390/plants9010099

**Published:** 2020-01-13

**Authors:** Paulina Ballesta, David Bush, Fabyano Fonseca Silva, Freddy Mora

**Affiliations:** 1 Institute of Biological Sciences, University of Talca, 2 Norte 685, Talca 3460000, Chile; pballesta@utalca.cl; 2 CSIRO–Australian Tree Seed Centre, Acton 2601, Australia; David.Bush@csiro.au; 3 Department of Animal Science, Universidade Federal de Viçosa, Viçosa 36570-900, Brazil; fabyanofonseca@ufv.br

**Keywords:** Bayesian models, deviance information criterion, marker-trait associations, predictive ability

## Abstract

High-throughput genotyping techniques have enabled large-scale genomic analysis to precisely predict complex traits in many plant species. However, not all species can be well represented in commercial SNP (single nucleotide polymorphism) arrays. In this study, a high-density SNP array (60 K) developed for commercial *Eucalyptus* was used to genotype a breeding population of *Eucalyptus cladocalyx*, yielding only ~3.9 K informative SNPs. Traditional Bayesian genomic models were investigated to predict flowering, stem quality and growth traits by considering the following effects: (i) polygenic background and all informative markers (GS model) and (ii) polygenic background, QTL-genotype effects (determined by GWAS) and SNP markers that were not associated with any trait (GSq model). The estimates of pedigree-based heritability and genomic heritability varied from 0.08 to 0.34 and 0.002 to 0.5, respectively, whereas the predictive ability varied from 0.19 (GS) and 0.45 (GSq). The GSq approach outperformed GS models in terms of predictive ability when the proportion of the variance explained by the significant marker-trait associations was higher than those explained by the polygenic background and non-significant markers. This approach can be particularly useful for plant/tree species poorly represented in the high-density SNP arrays, developed for economically important species, or when high-density marker panels are not available.

## 1. Introduction

A major focus of modern quantitative genetics is on assessing the association between polymorphic markers with phenotypic variations of complex traits. In this sense, genotype–phenotype studies for quantitative traits at the genome level usually require high-density genetic marker panels, i.e., a large number of markers throughout the genome and large population sizes to obtain sufficient power and prediction resolution [1,2]. The development of several genotyping platforms through high-density single nucleotide polymorphism (SNP) arrays, such as genotyping-by-sequencing (GBS) or SNP chips, has enabled the identification of quantitative trait loci (QTL) for different target traits in various plant species [3,4,5,6]. Silva-Junior et al. [7], for instance, developed a genome-wide SNP chip for multiple species of *Eucalyptus*, which has been effective for genomic studies in a wide variety of economically important eucalypt species and their hybrids, including *Eucalyptus grandis*, *Eucalyptus urophylla*, *Eucalyptus nitens* and *Eucalyptus globulus* [8,9,10,11,12]. However, despite the versatility of this SNP array, it does not perform as well in terms of genome coverage or number of available SNPs for species which are more-distantly related to those for which the chip was developed [13].

According to Pryce et al. [14], the use of low-density marker panels will inevitably affect the precision of QTL detection in genome-wide association studies (GWAS) and the accuracy of genomic prediction of target traits to some degree. On the other hand, Müller et al. [15] found that prediction models using a low-density marker panel (a subset of ~5000 SNPs) provided predictive abilities almost equivalent to using all available SNPs in *Eucalyptus* spp., however, they concluded that it is not yet clear whether the use of smaller SNP subsets is warranted for the long-term implementation of genomic selection in *Eucalyptus*, an aspect that remains still unknown. Marker panels could be considered as either low- or high-density depending on the genome size, the extent of linkage disequilibrium (LD) and the traits of interest. Larger genomes and rapid breakdown of LD would imply that a higher density of SNP loci would be needed to detect QTL. However, many authors quite arbitrarily describe panels as high or low density such that comparisons among organisms should be carefully made. In cattle (genome size 3 Gb), for instance, a DNA array of 50 K SNPs is considered a low-density panel [14], while in *Eucalyptus nitens* (640 Mb) the 60 K SNP chip was considered as a high-density marker panel by Suontama et al. [11]. However, LD in the domesticated bovine genome, though it decays rapidly [16], is more extensive than in nearly-wild eucalypts, thus comparison of SNP density is further complicated. Müller et al. [15] considered the subset of ~5 K SNPs that they used as a low-density marker panel in *Eucalyptus benthamii* and *Eucalyptus pellita*. Notably, low-density SNP chips are considered as a way to reduce the cost of high-density SNP panels in animal breeding and would enable cost-effective implementation of genomic studies [17,18]. In accordance with this, recently Silva et al. [19] used linkage (LA) and linkage disequilibrium (LDA) analyses (termed ‘LALDA’) for low density-based genomic selection (GS) purposes in animal breeding. In a Bayesian framework, the authors evaluated several GS models and verified the slight superiority of the LALDA models in comparison to traditional LDA models, concluding that the best performance evidenced by the LALDA approach can be due mainly to the small number of markers used, since it enabled to exploit relevant genomic regions that were not directly considered in the LDA, in which this extra information may have contributed to the improvement of the model performance. Similarly, other studies have explored the benefits of including fixed-effect covariates tagging peak genome-wide association study (GWAS) signals [20]. Based on their results, the authors conclude that the universal implementation of GS + GWAS for predicting the breeding values of all possible traits should be investigated on a trait-by-trait basis. On the other hand, Bernardo [21] determined whether explicitly modeling the effects of known major genes affects the response to genomic selection and showed that specifying a fixed effect for a single major gene was never disadvantageous except with a gene explains <10% of genetic variance. In that case, it should be included as a covariate in the traditional ridge-regression best linear unbiased prediction (RR-BLUP) model. Therefore, the objectives of this study were (i) to investigate the possible benefits of including marker-trait associations (from a GWAS analysis) and pedigree information into traditional GS model to predict complex traits in trees of *E. cladocalyx* using low-density SNP markers, and (ii) to assess the efficiency of Bayesian whole-genome regression models (Bayes A, Bayes B, Bayes Cπ and Bayesian Ridge Regression) in terms of predictive ability of complex traits and goodness-of-fit measures in the presence of QTL-genotype effects obtained from a marker-trait association analysis.

## 2. Results

### 2.1. SNP Data and Comparison of Genomic Prediction Models

In the present study, we genotyped a breeding population of *E. cladocalyx* using the 60 K SNP chip, yielding a subset of only ~3.9 K informative SNPs, due to the low number of polymorphic loci found at locations where they might have been expected. The ~3.9 K SNPs (~6% of the total SNP array) that were retained after filtering for minor allele frequency (MAF) and missing data were located in all eleven chromosomes of *Eucalyptus*, with an average of ~353 SNPs per chromosome, a density of 6 SNPs per 1 Mb and distributed with an average distance between SNPs of 11,600 bp (Table S1 and Figure S1). The genetic diversity of the genotyped population (evaluated with the 3879 SNPs) was 0.28 (in terms of expected heterozygosity) and the observed heterozygosity was 0.22. According to the population structure analysis, the studied population was strongly differentiated in three groups, with the following pairwise F_ST_ values: F_ST_ 1 = 0.086, F_ST_ 2 = 0.28 and F_ST_ 3 = 0.25.

**Figure 1:**
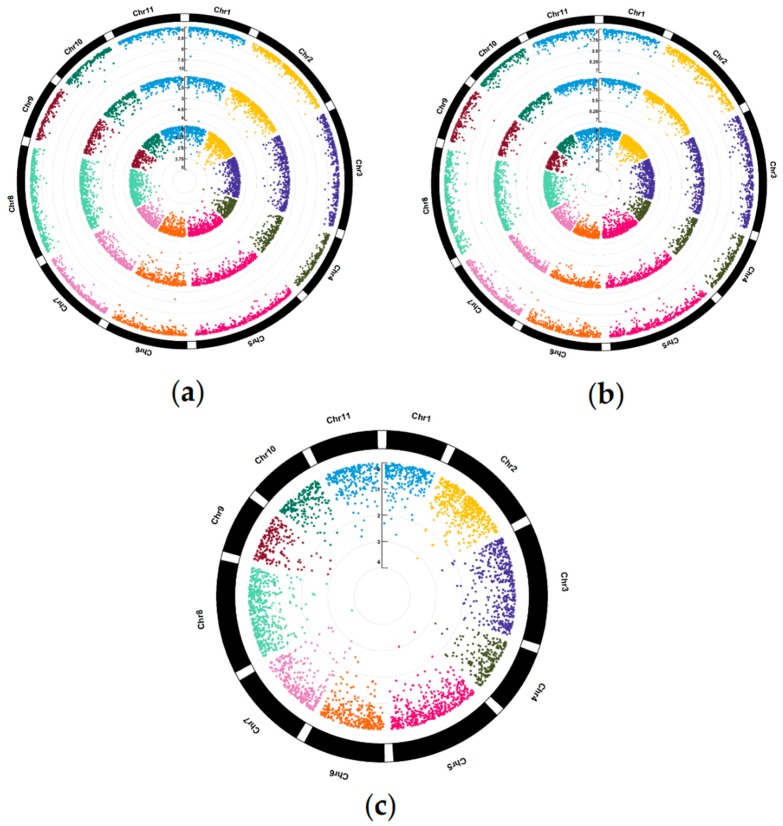
Manhattan plot for (**a**) growth-related traits (tree height, diameter at breast height and slenderness index; displayed from inside to outside), (**b**) stem quality traits (stem straightness, wood density and first bifurcation height; displayed from inside to outside) and (**c**) flowering intensity.

The association analysis identified a total of 90 significant marker-trait associations (MTAs), which were also distributed across all 11 chromosomes, of which 11, 16, 5, 26, 10, 5 and 17 MTAs were identified for total tree height (HT), diameter at breast height (DBH), stem straightness (STR), slenderness index (SLD), wood density (WD), flowering intensity (FI) and first bifurcation height (BHT), respectively (Figure 1). The MTAs explained a relatively low proportion of the total phenotypic variation, with values of 2% to 4%, 3% to 6%, 3% to 4%, 3% to 5%, 2% to 10%, 3% to 7% and 3% to 4% for HT, DBH, STR, BHT, SLD, WD and FI, respectively. The SNPs involved in these MTAs were posteriorly considered as relationship matrices in the prediction models that include the QTL-genotype effects (GSq model).

The prediction models that include the MTA information (GSq) and traditional genomic prediction models (GS) were compared in terms of goodness of fit through the Deviance Information Criterion (DIC) and predictive ability (PA). The DIC and PA values of all fitted models are shown in Table 1 and Table 2, respectively. GSq models outperformed the GS model in terms of PA when more than 10 significant MTAs were included in GSq predictions (i.e., the following traits HT, DBH, SLD, BHT and WD), whose values ranged between 0.19–0.39 (GS) and 0-24-0.45 (GSq). This result was consistent with the goodness-of-fit measures in most of cases. For HT, GS model outperformed the GSq model for Bayes B (BB), according to the ΔDIC value, while the GSq model was significantly superior in Bayes A (BA), Bayes C (BC) and Bayesian Ridge Regression (BRR) methods (ΔDIC > 10). The GSq approach had a higher predictive ability than the GS approach for all Bayesian models. For DBH, in contrast, the best performance (in terms of goodness-of-fit and PA) was obtained by GSq models based on any Bayesian prediction method (BA, BB, BC or BRR; with ΔDIC > 50). In addition, the predictive ability of DBH based on GSq model was two times higher than those based on traditional GS. For STR, GS models presented significantly lower DIC values than GSq models for BC and BRR (ΔDIC > 10), while the PA values for both approaches were similar. For SLD, goodness-of-fit measures for GSq models were better than traditional models based on any Bayesian genomic model (ΔDIC > 20). The predictive ability of SLD varied between 0.20–0.21 and 0.31–0.32 for GS and GSq models, respectively. For WD, the most of GSq models had better goodness-of-fit measures compared with GS models (ΔDIC > 5) for all Bayesian methods. The PAs of WD ranged between 0.27 and 0.43, which were higher for all GSq models than those based on the GS approach. For FI, GS models presented lower DIC values than GSq models for BA, BB, BC and BRR prediction methods (ΔDIC > 5). Predictive ability values for FI based on both approaches were similar and varied from 0.23 to 0.25. For BHT, there was a strong superiority of the GSq models over GS in all Bayesian methods in terms of goodness-of-fit measures (ΔDIC > 60). Moreover, the PA of BHT varied between 0.19–0.20 and 0.38–0.39 for GS and GSq models, respectively. Consistently with ΔDIC values, the predictive ability of BHT based on GSq models was two times higher than those based on GS models.

### 2.2. Heritability Estimates

The heritability estimates of the studied traits based on all genomic prediction models are shown in Table 3. The estimates of pedigree-based heritability (${\hat{h}}_{a}^{2}$) for GS were higher than GSq for all traits, whereas the values of genomic heritability (${\hat{h}}_{m}^{2}$ and ${\hat{h}}_{q}^{2}$) were dependent on the Bayesian prediction method for both models (GS and GSq). Based on the best fitted models (in terms of ΔDIC values), the heritability estimates of HT varied between 0.13 and 0.21 (${\hat{h}}_{a}^{2}$), 0.14 and 0.45 (${\hat{h}}_{m}^{2}$), and 0.29 and 0.34 (${\hat{h}}_{q}^{2}$). Based on the models with the lowest DIC values, the heritability estimates of DBH based on pedigree and MTAs (${\hat{h}}_{q}^{2}$) were similar, which ranged between 0.05 and 0.17, while the heritability estimates based on SNPs (${\hat{h}}_{m}^{2}$) varied between 0.4 and 0.45. In the case of STR, the estimates of pedigree-based heritability for GS models varied between 0.16 and 0.23, while the estimates of genomic heritability ranged between 0.3 and 0.32. For SLD, the heritability estimates based on pedigree information varied between 0.08 and 0.10. The heritability estimates based on MTAs varied between 0.33 and 0.39, while the heritability estimates based on SNPs (${\hat{h}}_{m}^{2}$) varied between 0.02 and 0.17. The heritability estimates of WD based on pedigree information (GSq models) ranged between 0.14 and 0.17. The heritability estimates of no-significant QTLs by MTAs analysis varied between 0.12 and 0.24, and those for significant QTLs ranged between 0.27 and 0.31. The heritability estimates of FI based on pedigree with a better goodness-of-fit measure (i.e., the GS model) varied between 0.27 and 0.34, while the genomic heritability estimates ranged between 0.07 and 0.29. Based on the models with the lowest DIC values (i.e., all GSq models), the pedigree-based heritability of BHT was 0.08 in the context of all Bayesian methods (BA, BB, BC and BRR). The heritability estimates based on non-significant SNPs by MTAs analysis varied between 0.04 and 0.13, while those based on the significant QTL varied between 0.41 and 0.45.

## 3. Discussion

### 3.1. Marker-Trait Associations for All Studied Traits

*Eucalyptus cladocalyx* is not a close relative of other eucalypts. Brooker [22] placed *E. cladocalyx* in the monophyletic section *Sejunctae* [23]. As the marker panel we used had been developed for the most widely-planted species which all fall within sections *Maidenaria*, *Exsertaria* and *Latoangulatae*, a lower rate of cross-species amplification of SSR (Simple Sequence Repeats) can be expected in *E. cladocalyx* [24] due to differentiation among widely-distant sections. Despite the low availability of SNP markers, the genetic diversity values were similar to other previous studies of natural populations of *E. cladocalyx* [23,25]. The strong genetic differentiation in three clusters had also been previously reported [23,25,26].

Ninety significant marker-trait associations (MTAs) were detected for the seven target traits, which were subsequently used for the GSq approach. In accordance with this, several studies have previously identified genomic regions explaining part of the phenotypic variation of growth-related traits in *E. cladocalyx* (e.g., Ballesta et al. [25], Arriagada et al. [26], Maldonado et al. [27], Valenzuela et al. [28]). For HT, DBH and SLD, the MTAs were mainly located on chromosomes Chr2 (5 and 4 MTAs for HT and DBH, respectively), Chr6 (3 and 6 MTAs for DBH and SLD, respectively), Chr8 (4 MTAs for SLD) and Chr10 (4 MTAs for SLD). In agreement with these results, Maldonado et al. [27] detected one QTL, based on Simple Sequence Repeat (SSR) markers, located on linkage group LG6 explaining up to 27% of the phenotypic variation of DBH. In addition, Arriagada et al. [26] reported SSR markers associated with HT and DBH, located on the linkage groups LG6, LG8 and LG10, explaining up to 23% of the phenotypic variation.

Flowering components are target traits in breeding programs of some species of *Eucalyptus*, as the flowers provide a reliable source for honey production [26,29]. In dry regions of Chile and South Africa, prolific flowering from forest plantations of *E. cladocalyx* and other eucalypts is particularly advantageous for the supply of honey [30]. In the present study, almost all associations (4/5 MTAs) for flowering intensity were located on chromosome Chr2, which is in accordance with Missiaggia et al. [31], who reported a major QTL located on chromosome Chr2 (*Eef1*) controlling the early flowering in *Eucalyptus grandis*. In *E. cladocalyx*, previous studies have reported that the flowering intensity and early flowering have a positive genetic correlation and common QTLs controlling the phenotypic variation of both traits [29,32]. Interestingly, according to linkage disequilibrium analyses, only two significant SNPs (MTAs for FI) were in disequilibrium, which covered a genomic region of 17,849 bp. For WD, BHT and STR, the MTAs were mainly located on chromosomes Chr2 (WD), Chr5 (BHT) and Chr8 (STR, WD and BHT). In accordance whit this, Valenzuela et al. [28] detected a QTL located on Chr2 explaining 8% of the phenotypic variation of WD in *E. cladocalyx*.

### 3.2. Comparison between Genomic Prediction Models

Several studies have explored the potential of the selection based on genomic tools in forest species [33,34,35,36], including *E. cladocalyx* [12,37,38]. According to the results, GSq models outperformed traditional GS models in terms of predictive ability when at least ten significant marker-trait associations were included in GSq. In addition, another important finding of this study was that the GS and GSq models that include the pedigree information (i.e., pedigree information as a relationship matrix), outperformed the model based solely on SNP marker effects (in terms of goodness-of-fit), revealing the importance of polygenic effects in the prediction model based on low-density markers; an aspect emphasized by Silva et al. [19]. For instance, the predictive ability of flowering intensity based on only the SNP marker panel was three times lower than those based on GS or GSq.

According to De Los Campos et al. [39], Bayes A, Bayes B, Bayes C and BRR methods can improve the predictive ability in genome-based evaluations, but these prediction models could have overfitting problems when the ratio of number of markers and individuals is over 50 [40]. To overcome this, the use of genomic relationship matrices between individuals into genomic prediction models could beneficially capture general information and reduce the dimensionally problem [41,42], while exploring regions in linkage disequilibrium with QTLs [43]. Interestingly, we found that the GSq models had a better fit than GS models and, at the same time increased the predictive ability of BHT, DBH, SLD and WD. Notably, these benefits were only detected for the prediction of traits with greater than ten MTAs, while for the traits with a lower number of MTAs (i.e., HT, FI and STR), the GS models had a better fit than GSq models. The predictive ability for STR and FI was similar between GSq and GS models. It is worth mentioning that the MTAs detected by the classical linkage analysis explained relatively low values of the total phenotypic variation; which is in accordance with the genetic architecture of quantitative traits. According to Silva et al. [19], the superior performance of the models that include QTL information compared with GS model can be due to GSq model exploiting relevant regions not directly considered in GS model, improving the performance of the prediction model. Additionally, previous studies have reported that a pre-selection of SNPs or the use of genome-wide association analyses to identify and rank markers could increase predictive ability [38,44,45].

Although the analytical assumptions differ among studied Bayesian genomic models, the predictive ability of the studied traits was not severely different among them. In accordance with our findings, several studies have reported that the predictive ability did not differ between methods in forest tree species, especially for growth and wood quality related traits [15,33,36,46,47,48]. In the present study, the main differences in predictive ability values were observed between the GSq and GS methods, so that the superiority of GSq (or GS) to predict the studied traits was conserved for any Bayesian genomic model.

### 3.3. Heritability Estimates

According to the goodness-of-fit measures, the GSq model outperformed the GS model for BHT, DBH, SLD and WD, whereas GS offered better model fit compared with GSq for STR and FI. Additionally, for HT, DBH, SLD, WD and BHT, the genomic heritability estimates based on MTAs were higher than the estimates based on SNPs (not significantly associated with a trait). On the other hand, the genomic heritability estimates based on SNP markers (not associated) considering BB and BC method, were higher than those based on BA and BRR methods (for all traits, except HT). BB and BC models involve variable selection procedures, which favor the selection based on major effect markers/genes [49]. Notably, other studies have confirmed that some whole-regression methods could overestimate the heritability values [50], and therefore, these findings should be interpreted with caution. Overall, all target traits had pedigree-based heritability estimates from low to moderate (h^2^ = 0.08–0.34), which are in accordance with the range usually expected for forest tree growth, flowering and stem quality related traits, including *E. cladocalyx* [28,29,51,52,53,54].

The heritability estimates based on MTAs for STR and FI were lower than those based on pedigree information or non-significant SNPs from the MTA analysis. In tree species, several studies had reported genomic regions explaining a high percent of phenotypic variation of STR and FI. For example, Arriagada et al. [26] reported one QTL explaining up to 15% of the total variation of STR in *E. cladocalyx*. In a meta-analysis, Hall et al. [55] confirmed that phenological traits are highly heritable and controlled by key genomic regions in trees. In fact, Missiaggia et al. [31] reported a key region on chromosome Chr2 (*Eef1*) controlling the early flowering in *E. grandis*. In this context, low-density SNP panels could limit the probability to detect key regions explaining the phenotypic variation of these traits. In addition, these results are supported by the fact that the GS model outperformed the GSq model for STR and FI (in terms of goodness-of-fit), which means that the total variation of STR and FI is better explained by the classic prediction model (i.e., $\sigma_{a}^{2}\;$ and $\;\sigma_{m}^{2}$).

## 4. Materials and Methods

### 4.1. Plant Material and Phenotypic Evaluation

A genomic selection study was performed in a long-term open-pollinated progeny trial comprising 49 families of *E. cladocalyx* established in 2001 in northern Chile (locality of Los Vilos; 31°54′ S; 71°27′ W; 167 m.a.s.l). The climate is classified as predominantly arid, according to the De Martonne aridity index [26]. Trees were arranged in a randomized complete block design with 30 blocks and single-tree plots (total of 1470 trees). Trees were planted at 2 m spacing within rows and 3 m between rows (~1667 trees ha^−1^). The following quantitative traits were measured in 17 years-old trees: diameter at breast height (DBH), total tree height (HT), first bifurcation height (BHT), wood density (WD), stem straightness (STR) and slenderness index (SLD). The BHT was rated on a scale of five levels, in which a value of 1 is assigned to trees with a loss of the central axis in the first fifth of the tree’s height, a value of 2 indicates that a loss of the central axis occurs in the second fifth of the tree, a value of 3 indicates that a loss of the central axis occurs in the third fifth of the tree, a value of 4 implies a loss of the central axis in the fourth fifth of the tree’s height, and a value of 5 implies that a loss of the central axis in the last fifth of the tree height or does not show loss of the apical axis (modified scale by Bush et al. [54]). WD was measured indirectly according to Valenzuela et al. [28]. STR was measured in the first two-thirds of the total height of tree and was considered as ordinal variables with four levels [53], in which a value of 0 if the stem was strongly twisted, a value of 1 if the stem presents moderate levels of curvature, a value of 2 if the stem was slightly curved, and 3 if the stem was completely straight. The SLD was calculated as the ratio between HT (m) and DBH (m). Additionally, flowering intensity (FI) was measured using a scale that ranged from 0 to 3, in 18 years-old trees, according to Arriagada et al. [26], where a value of 0 means absence of flowers, buds and/or capsules, a value of 1 sparse flowers on a small part of crown, a value of 2 if the flowers/capsules/buds were covering the half of the crown, and a value of 3 for trees with numerous flowers on the whole crown.

### 4.2. DNA Extraction and Tree Genotyping

DNA for tree genotyping was isolated from leaf tissues of 480 individuals according to Porebsky et al. [56] and Doyle and Doyle [57]. On average, 10 individuals per family were randomly selected to be genotyped using the Illumina Infinium EUChip60K SNP array [7]. The SNP data were filtered for SNP call rate score >0.7 and minor allele frequency (MAF) >0.05. The missing data were imputed using the LD-kNNi method in Tassel 5.2 [58]. Linkage disequilibrium between marker pairs (MTAs at the same chromosome) was calculated using TASSEL version 5.2.

### 4.3. Genomic Prediction Models

The following four Bayesian whole-genome regression models were used for the estimation of SNP marker effects, variance components and genomic heritability: Bayes A ([59]; BA), Bayes B ([59]; BB), Bayes Cπ ([60]; BC) and Bayesian Ridge Regression ([61]; BRR). Due to the low density of markers found in this studied population, the following two approaches were used to predict the studied traits: traditional genomic prediction model (GS), considering all informative markers (~3.8 K SNP) from the commercial SNP panel, and a combination of traditional GS and QTL information (GSq). The GS model is defined as:

(1) $$y*=1\mu +\overset{m}{\underset{i=1}{\sum }}x_{i}m_{i}+Za+\varepsilon$$

where y* is the vector of phenotypic records pre-corrected for the effects of block and genetic structure [62,63]. Respectively, **1** and μ are vectors of ones and overall mean. $m_{i}$ corresponds to the additive genetic effect of the *i*-th marker, with m as the number of markers. $x_{i}$ is the incidence vector of each marker (codified as: AA = 0, AB = 1 and BB = 2). **a** corresponds to polygenic effects, with **a**~**N**(0, ${\hat{\sigma }}_{a}^{2}$ **A**), and **Z** is the incidence matrix related to polygenic effects. The coefficients of relationship matrix were adjusted according to Bush et al. [54]. Finally, ε is the residual vector, ε~**N**(0, ${\hat{\sigma }}_{e}^{2}$ **I**_n_). The genomic predictions were performed using BGLR package in R [64]. Specifically, 1,000,000 iterations of Markov Chain Monte Carlo simulations were used in all genomic prediction models, with a burn-in period of 100,000.

The GSq model consisted of two steps, in which the first step involves a marker-trait association (MTA) analysis for QTL detection according to Silva et al. [19]. The MTA analysis was conducted using the following mixed linear model:

(2) y*=Sa+Qv+Zu+ε

where y* is a vector of adjusted phenotypic observations (by the block effect). **S**, **Q** and **Z** correspond to the incidence matrices for **a**, **v** and **u**, respectively. **a** and **v** are the vectors of SNP effects (fixed) and population structure effects (fixed). **u** corresponds to a vector of polygenic effects (random), and ε is the residual vector. The variances of **u** and ε are $\mathrm{Var}\left(u\right)=2K\sigma_{g}^{2}$ and $\mathrm{Var}\left(\varepsilon \right)=R\sigma_{e}^{2}$, respectively, where **K** is the kinship coefficient matrix, which was estimated using the program TASSEL [58]. For this analysis, the significance threshold of 0.001 and a false discovery rate (FDR) <10% were applied to test for significant MTAs according to Uchiyama et al. [65]. The GWAS analysis was carried out using rrBLUP package in R v. 3.5 [66]. The population structure was assessed by Bayesian model-based clustering in STRUCTURE software v. 2.3.4 [67]. In addition, the SNP data was used to estimate the genetic diversity of the genotyped population using GenAlex v.6.5 [68].

The second step of the GSq approach consists of the compilation of traditional GS model (Equation (1)) and QTL-genotype effect (**q**) from the association analysis, which is expressed as:

(3) $$y*=1\mu +\overset{m}{\underset{i=1}{\sum }}x_{i}m_{i}+Za+Zq+\varepsilon$$

where y* is the vector of phenotypic records pre-corrected for the effects of block and genetic structure, and **1** and μ are vectors of ones and overall mean, respectively. $m_{i}$ corresponds to the additive genetic effect of the *i*-th marker that were not found to be significantly associated with a trait. $x_{i}$ is the incidence vector of each marker (AA = 0, AB = 1 and BB = 2). The variance of m (${\hat{\sigma }}_{m}^{2}$) depends on the Bayesian model implemented. **Z** is the incidence matrix of polygenic (**a**) and QTL-genotype (**q**) effects assuming **a**~**N**(0, ${\hat{\sigma }}_{a}^{2}$**A**) and **q**~**N**(0, ${\hat{\sigma }}_{q}^{2}$**Q**), respectively. The **Q** is a covariance matrix, in which the elements are the probabilities that individuals are identical by descent based on significant SNP markers according to the MTA analysis.

### 4.4. Heritability Estimates

For the GSq model, the heritability estimates were obtained as follows:

(4) $${\hat{h}}_{a}^{2}=\frac{{\hat{\sigma }}_{a}^{2}}{{\hat{\sigma }}_{a}^{2}+{\hat{\sigma }}_{m}^{2}+{\hat{\sigma }}_{q}^{2}+{\hat{\sigma }}_{e}^{2}}$$

(5) $${\hat{h}}_{m}^{2}=\frac{{\hat{\sigma }}_{m}^{2}}{{\hat{\sigma }}_{a}^{2}+{\hat{\sigma }}_{m}^{2}+{\hat{\sigma }}_{q}^{2}+{\hat{\sigma }}_{e}^{2}}$$

(6) $${\hat{h}}_{q}^{2}=\frac{{\hat{\sigma }}_{q}^{2}}{{\hat{\sigma }}_{a}^{2}+{\hat{\sigma }}_{m}^{2}+{\hat{\sigma }}_{q}^{2}+{\hat{\sigma }}_{e}^{2}}$$

where ${\hat{h}}_{a}^{2}$, ${\hat{h}}_{m}^{2}$ and ${\hat{h}}_{q}^{2}$ correspond to heritability estimates based on pedigree information, a set of markers that were not found to be significantly associated with a trait and a set of SNPs significantly associated with a trait (MTAs), respectively. ${\hat{\sigma }}_{a}^{2}$ is the variance due to the additive polygenic effect, ${\hat{\sigma }}_{m}^{2}$ corresponds to the marker effect variance, ${\hat{\sigma }}_{q}^{2}$ is the variance of markers significantly associated with a trait, and ${\hat{\sigma }}_{e}^{2}$ is the residual variance.

For traditional GS model, the term ${\hat{h}}_{m}^{2}$ correspond to the heritability estimates based on all SNPs (n = 3879). In the cases of BC and BRR methods, ${\hat{\sigma }}_{m}^{2}$ was calculated as: ${\hat{\sigma }}_{m}^{2}=2{\hat{\sigma }}_{SNP}^{2}\overset{n}{\underset{i=1}{\sum }}{\hat{p}}_{i}(1-{\hat{p}}_{i})$, in which ${\hat{\sigma }}_{SNP}^{2}$ corresponds to a common variance for SNP markers and ${\hat{p}}_{i}$ is the MAF of the *i*-th marker. The ${\hat{\sigma }}_{m}^{2}$ term for BA and BB models was estimated as ${\hat{\sigma }}_{m}^{2}=2\overset{n}{\underset{i=1}{\sum }}{\hat{p}}_{i}(1-{\hat{p}}_{i}){\hat{\sigma }}_{SNP_{i}}^{2}$, in which ${\hat{\sigma }}_{SNP_{i}}^{2}$ corresponds to the variance due to the *i*-th marker.

### 4.5. Comparison between Genomic Prediction Models

The Bayesian prediction models were compared in terms of goodness-of-fit, by using Deviance Information Criterion (DIC) [69], and the predictive ability (PA). The DIC is defined by the following expression:

(7) $$DIC=\bar{D}+pD$$

where $\overset{¯}{D}$ is a Bayesian measure of model fit, which is defined as the posterior expectation of the deviance $\left(\bar{D}=E_{\theta /y}\left[-2\cdot lnf\left(y/\theta \right)\right]\right)$; *pD* is the effective number of parameters. A *DIC* difference of more than 10 between two competitive models (GSq and GS models) was considered to be supported against a model with higher *DIC*; a *DIC* difference between 5 and 10 was considered as substantial difference between models, and a difference less than 5 was considered as not significant.

The predictive ability of each model was calculated as the correlation between the pre-corrected observations (y*) from validation dataset and the estimated breeding value (${\hat{y}}^{\;*}$). A total of 20-fold cross-validation was used to evaluate the predictive ability of all models. The ${\hat{y}}^{\;*}$ for GS and GSq models was calculated as ${\hat{y}}^{\;*}=\overset{n}{\underset{i=1}{\sum }}x_{i}{\hat{m}}_{i}+Z\hat{u}$ and ${\hat{y}}^{\;*}=\sum_{i=1}^{n}x_{i}{\hat{m}}_{i}+Z\hat{u}+Z\hat{q}$, respectively.

## 5. Conclusions

In this study, we evaluated the performance of Bayesian genomic models that include the genetic background (pedigree) and QTL information from GWAS analysis, which was specially implemented in the context of a low-density SNP markers. Importantly, predictive abilities were superior in GSq when the proportion of the variance explained by the significant marker-trait associations (MTAs) was higher than those explained by the polygenic background and SNP markers (that were not found to be significantly associated with a trait). Therefore, we emphasized and hypothesized that both the number of associations and/or the percentage of variation explained by the MTAs are determinants in the effectiveness of the GSq method. This approach can be particularly useful for plant/tree species poorly represented in the high-density SNP arrays, developed for economically important species or when high -density marker panels are not available.

## Figures and Tables

**Table 1:** Deviance information criterion (DIC) of genomic prediction in *Eucalyptus cladocalyx* based on (i) polygenic background (pedigree information) and all informative markers (GS model) and (ii) polygenic background, QTL-genotype effects (determined by GWAS) and SNP markers that were not associated with any trait (GSq model).

Trait/Model	Bayes A	Bayes B	Bayes C	BRR ^b^
Tree height				
GS	1968.9	1959.5	1968.6	1965.4
GSq	1951.2	1971.3	1941.3	1941.2
ΔDIC ^a^	17.7 **	11.8 **	27.3 **	24.2 **
Diameter at breast height				
GS	2556.5	2544.7	2539.8	2538.2
GSq	2490.4	2480.7	2480.2	2473.3
ΔDIC	66.1 **	64.0 **	59.6 **	64.9 **
Stem straightness				
GS	947.7	941.4	932.7	935.3
GSq	947.0	944.7	947.2	946.2
ΔDIC	0.7	3.3	14.5 **	10.8 **
Slenderness index				
GS	4302.5	4299.5	4294.3	4290.5
GSq	4268.1	4268.5	4264.3	4261.3
ΔDIC	34.4 **	31.0 **	30.0 **	29.2 **
Wood density				
GS	2094.3	2082.4	2101.8	2042.0
GSq	2067.1	2075.9	2067.6	2070.3
ΔDIC	27.2 **	6.5 *	34.3 **	28.4 **
Flowering intensity				
GS	1293.9	1301.2	1282.3	1285.9
GSq	1306.0	1309.7	1301.3	1295.2
ΔDIC	12.1 **	8.4 *	19.0 **	9.4 *
First bifurcation height				
GS	1491.9	1490.6	1487.3	1485.8
GSq	1426.6	1424.9	1424.9	1423.5
ΔDIC	65.3 **	65.7 **	62.4 **	62.3 **

^a^ Difference between DIC values of GSq and GS models. ^b^ Bayesian Ridge Regression. * Substantial statistical difference between GSq and GS models. ** Strong evidence of statistical difference between GSq and GS models.

**Table 2:** Predictive ability (PA) of all studied traits in *Eucalyptus cladocalyx* according to (i) polygenic background (pedigree information) and all informative markers (GS model) and (ii) polygenic background, QTL-genotype effects (determined by GWAS) and SNP markers that were not associated with any trait (GSq model). The PA values for each method correspond to the mean of PA values for 20-folds of cross-validation.

Trait/Model	Bayes A	Bayes B	Bayes C	BRR ^a^	${{\overset{¯}{X}}_{PA}}^{b}$
Tree height					
GS	0.33	0.32	0.33	0.34	0.33
GSq	0.45	0.44	0.44	0.45	0.44
Diameter at breast height					
GS	0.21	0.23	0.22	0.22	0.22
GSq	0.41	0.41	0.41	0.42	0.41
Stem straightness					
GS	0.39	0.39	0.39	0.39	0.39
GSq	0.40	0.40	0.40	0.39	0.40
Slenderness index					
GS	0.20	0.20	0.21	0.21	0.21
GSq	0.32	0.32	0.31	0.31	0.32
Wood density					
GS	0.27	0.27	0.27	0.28	0.27
GSq	0.43	0.43	0.43	0.43	0.43
Flowering intensity					
GS	0.25	0.25	0.24	0.23	0.24
GSq	0.25	0.25	0.25	0.24	0.25
First bifurcation height					
GS	0.19	0.20	0.20	0.19	0.19
GSq	0.38	0.38	0.39	0.39	0.38

^a^ Bayesian Ridge Regression. ^b^ Corresponds to the average of PA values.

**Table 3:** Estimates of heritability of the studied traits for each Bayesian genomic model and effect (i) polygenic background (pedigree information) and all informative markers (GS model), and (ii) polygenic background, QTL-genotype effects (determined by GWAS) and SNP markers that were not associated with any trait (GSq model). ${\hat{h}}_{a}^{2}$ corresponds to the pedigree-based estimated heritability. ${\hat{h}}_{m}^{2}$ is the heritability estimate based on a set of markers that were not found to be significantly associated with a trait (GSq) or all SNP markers (GS), ${\hat{h}}_{q}^{2}$ represents the heritability estimates based on a set of SNPs significantly associated with a trait.

Trait/Model	Bayes A			Bayes B			Bayes C			BRR ^a^		
	${\hat{h}}_{a}^{2}$	${\hat{h}}_{m}^{2}$	${\hat{h}}_{q}^{2}$	${\hat{h}}_{a}^{2}$	${\hat{h}}_{m}^{2}$	${\hat{h}}_{q}^{2}$	${\hat{h}}_{a}^{2}$	${\hat{h}}_{m}^{2}$	${\hat{h}}_{q}^{2}$	${\hat{h}}_{a}^{2}$	${\hat{h}}_{m}^{2}$	${\hat{h}}_{q}^{2}$
Tree height												
GS	0.28	0.24	-	0.21	0.45	-	0.22	0.40	-	0.29	0.24	-
GSq	0.16	0.14	0.32	0.18	0.12	0.29	0.13	0.27	0.29	0.14	0.17	0.34
Diameter at breast height												
GS	0.20	0.14	-	0.15	0.37	-	0.15	0.39	-	0.19	0.24	-
GSq	0.11	0.05	0.44	0.09	0.15	0.42	0.09	0.17	0.40	0.10	0.11	0.45
Stem straightness												
GS	0.23	0.32	-	0.18	0.31	-	0.16	0.30	-	0.21	0.30	-
GSq	0.18	0.18	0.01	0.14	0.37	0.01	0.14	0.32	0.01	0.18	0.19	0.013
Slenderness index												
GS	0.19	0.12	-	0.16	0.27	-	0.15	0.33	-	0.18	0.21	-
GSq	0.09	0.02	0.39	0.08	0.05	0.36	0.08	0.17	0.33	0.09	0.10	0.35
Wood density												
GS	0.25	0.28	-	0.18	0.50	-	0.19	0.45	-	0.21	0.42	-
GSq	0.17	0.13	0.31	0.17	0.18	0.27	0.14	0.24	0.27	0.17	0.12	0.31
Flowering intensity												
GS	0.34	0.07	-	0.32	0.10	-	0.27	0.29	-	0.33	0.13	-
GSq	0.30	0.06	0.00	0.29	0.06	0.00	0.27	0.20	0.00	0.31	0.10	0.002
First bifurcation height												
GS	0.20	0.05	-	0.19	0.12	-	0.16	0.27	-	0.19	0.14	-
GSq	0.08	0.04	0.44	0.08	0.11	0.42	0.08	0.13	0.41	0.08	0.06	0.45

^a^ Bayesian Ridge Regression.

## Supplementary Materials

The following are available online at https://www.mdpi.com/2223-7747/9/1/99/s1, Figure S1: Ideogram representing the SNP density in a *Eucalyptus cladocalyx* population genotyped by the 60K SNP array, Table S1: Summary of the single nucleotide polymorphism (SNP) density in *Eucalyptus cladocalyx*.
