# Supplementary material for: Genomic Predictions Using Low-Density SNP Markers, Pedigree and GWAS Information: A Case Study with the Non-Model Species Eucalyptus cladocalyx
Source: Plants (Basel). 2020 Jan 13;9(1):99. doi: 10.3390/plants9010099 (PMC7020392; doi:10.3390/plants9010099)
Supplement: Supplementary file 1 [file plants-09-00099-s001.docx]

Supplementary Material

Genomic predictions using low-density SNP markers, pedigree and GWAS information: a case study with the non-model species *Eucalyptus cladocalyx*

Paulina Ballesta^1^; David Bush^2^; Fabyano Fonseca Silva^3^; Freddy Mora^1^*

^1^ Institute of Biological Sciences, University of Talca, 2 Norte 685, Talca 3460000, Chile; pballesta@utalca.cl

^2^ CSIRO–Australian Tree Seed Centre, Acton 2601, Australia; [David.Bush@csiro.au](mailto:David.Bush@csiro.au)

^3^ Department of Animal Science, Universidade Federal de Viçosa, Viçosa 36570-900, Brazil; fabyanofonseca@ufv.br

***** Correspondence: fmora@utalca.cl

Received: 25 November 2019; Accepted: 9 January 2020; Published: date

________________________________________________________________________________

**Table S1**. Summary of the single nucleotide polymorphism (SNP) density in *Eucalyptus cladocalyx*.

| **Chromosome** | **Total SNP (60K)** | **SNP–EC*** | **SNPs per 1Mb*** |
| --- | --- | --- | --- |
| 1 | 4227 | 246 | 6.1 |
| 2 | 6725 | 534 | 8.3 |
| 3 | 6560 | 484 | 6.1 |
| 4 | 4170 | 233 | 5.6 |
| 5 | 6335 | 482 | 6.5 |
| 6 | 6312 | 358 | 6.7 |
| 7 | 5100 | 312 | 6.0 |
| 8 | 7455 | 547 | 7.4 |
| 9 | 4147 | 205 | 5.3 |
| 10 | 4601 | 215 | 5.5 |
| 11 | 5096 | 263 | 5.9 |
| Total | 60728 | 3,879 | - |
| Mean | 5520.7 | 352.6 | 6.3 |

*SNPs found in *E. cladocalyx* considering a call rate score > 0.7 and MAF > 0.05


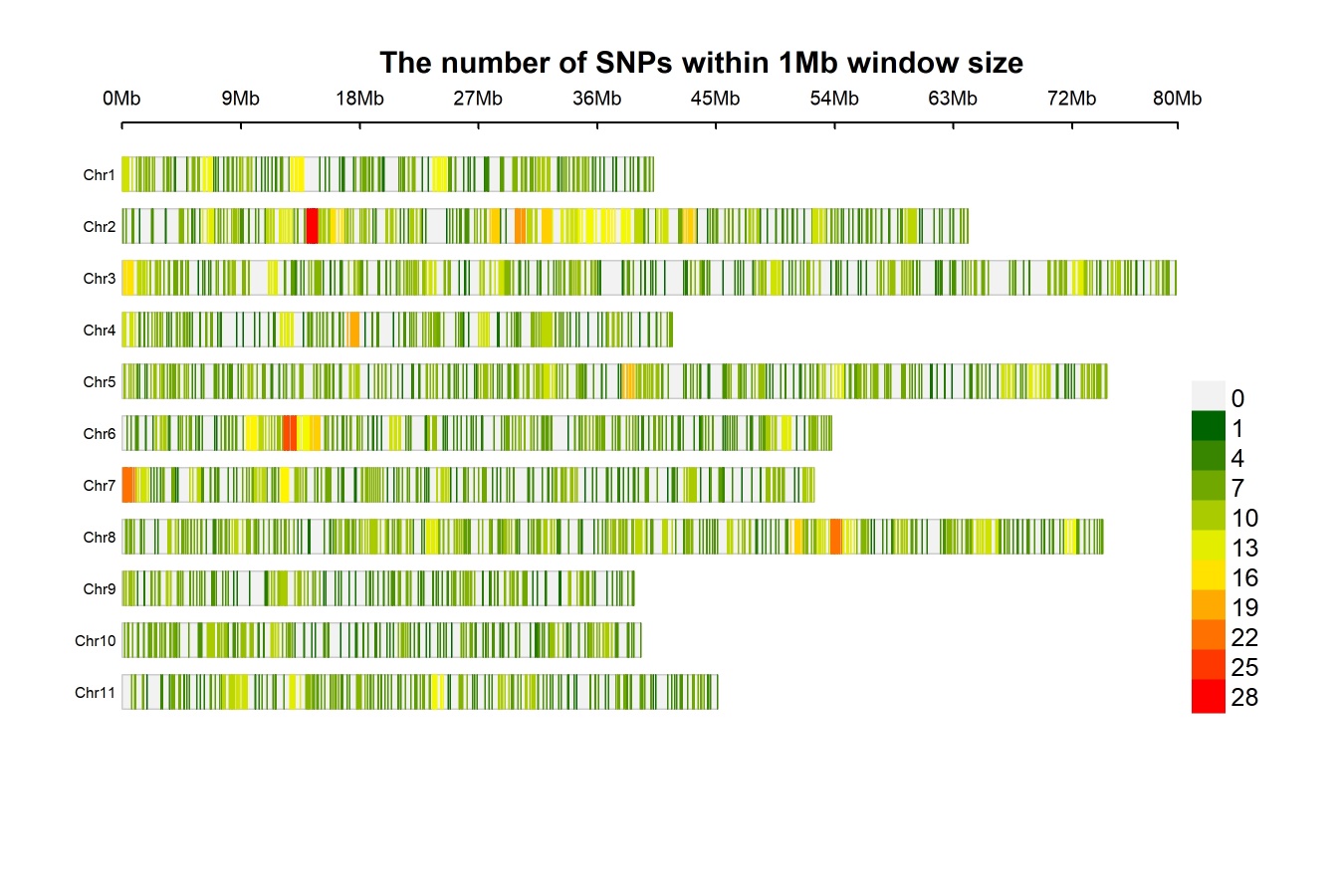


**Figure S1**. Ideogram representing the SNP density in a *Eucalyptus cladocalyx* population genotyped by the 60K SNP array.
